# 

**Published:** 1899-08

**Authors:** 


### CONTENTS.

ORIGINAL COMMUNICATIONS—

The Pathology of Fever. By J. Clarence Johnson, M.D., Atlanta, Ga.......................

Something of the Tact and Patience Necessary in the Prognosis and Treatment of Chronic Deafness. By Arthur G. Hobbs, M.D., Atlanta, Ga.......................

A Report of Two Interesting Cases. By Marcus F. Carson, M.D., Griffin, Ga.......................

Abdomino-Perineal Procto-Sigmoidectomy. By James N. Ellis, M.D., Atlanta, Ga.......................

SOCIETY REPORTS—

Atlanta Society of Medicine.......................

EDITORIAL NOTES AND COMMENTS—

Reading Character from Inspection of the Vulva and Vagina.......................

"A Gigantic Mistake".......................

CONTENTS CONTINUED.......................

